# Managing Diabetes Mellitus: A Survey of Attitudes and Practices Among Family Physicians

**DOI:** 10.1007/s10900-015-0024-2

**Published:** 2015-04-16

**Authors:** Yacov Fogelman, Margalit Goldfracht, Khaled Karkabi

**Affiliations:** Department of Family Practice, Leumit HMO and Rappaport Faculty of Medicine, Technion - Israel Institute of Technology, P.O. Box 121, 23800 Givat Elah, Israel; The Ruth and Bruce Rappaport Faculty of Medicine, Technion - Israel Institute of Technology, Haifa, Israel; Department of Family Medicine, Clalit Health Services, Haifa and Western Galilee District, Haifa, Israel

**Keywords:** Primary care physician, Diabetes, Survey, Family physician

## Abstract

Due to the increasing prevalence of diabetes and the shortage of endocrinologists, family physicians have an important role in diabetes management. The purpose of this study was to examine the sources of knowledge, attitudes and practices of family physicians regarding the management of type 2 diabetes. Attendees at continuous medical education (CME) programs in Israel were requested to respond anonymously to written questions about their sources of knowledge about diabetes, the methods of diabetes management they advise their patients, their knowledge of diabetes medication treatments, and their attitudes toward people with type 2 diabetes. Questionnaires were completed by 362 family physicians (79 % response rate). Of them, 329 (91 %) reported that they usually manage their patients’ diabetes care, including that of patients with concomitant risk factors. Their most common recommendations for diabetes control were: to increase physical activity, decrease total calorie intake, consult with a dietitian and undergo weight loss counseling. Almost all physicians (97 %) reported providing lifestyle change counseling. Sixty percent reported lacking knowledge about nutritional issues. Only 58 % answered correctly regarding the effect of the anti-diabetic drug, GLP1 analog. Board certified family physicians and their residents exhibited more knowledge about diabetes practice than did non-board certified family physicians. The great majority of family physicians surveyed usually manage their patients’ diabetes themselves, and do not refer them to diabetes specialists. The implementation of strategies that will enhance the competencies and confidence of family physicians in diabetes management are important for achieving successful treatment.

## Introduction

Type 2 diabetes mellitus is a major public health concern worldwide. Almost 10 % of the world’s population is predicted to have diabetes by 2030 [1, 2]. Lifestyle and pharmacologic interventions are claimed to halve the development of type 2 diabetes among individuals at high risk [3, 4]. During the past decade, Israel has become a part of the worldwide epidemic of obesity and diabetes. An Israeli population-based study reported a rise in the direct medical costs of diabetes in a health maintenance organization (HMO) setting [5]. A study of an urban population in the center of Israel showed prevalence rates of adult-onset diabetes as 21 % among Arabs and 12 % among Jews [6]. A 12-year follow-up of a population-based primary care diabetes program in Israel reported an increase in the prevalence of diabetes from 20.2/1000 in 1995 to 63.7/1000 in 2007 [7]. The high prevalence of diabetes and its substantial health-economic burden call for timely and efficient screening, diagnoses and management.

The number of adult endocrinologists in the United States is insufficient to meet current and future demands, according to a recently published workforce study [8]. In Israel, the waiting time for an appointment with an endocrinologist can be several weeks to several months. The majority of Israelis with diabetes are diagnosed, treated and managed by family physicians as front-line providers of care, rather than by diabetologists and endocrinologists [7]. Optimal care requires family physicians to be competent in the complexities of diabetes management, as well as in patient communication, counselling and education, since addressing patients’ psychological barriers to disease management is considered important for improving outcomes [9].

The family physician is in a unique position of influencing and treating people with diabetes, toward the adoption of lifestyle changes and the prevention of disease complications [10, 11]. However, family physicians confront numerous challenges when caring for people with diabetes, which are similar across international and health system borders [12, 13]. The aim of this study was to assess knowledge, attitudes and practices in diabetes management among Israeli family physicians in an era of rapidly growing diabetes prevalence.

## Methods

During 2012–2013, three investigators (YF, MG, KK) administered an anonymous questionnaire to family physicians participating in continuing medical education (CME) programs affiliated with all the academic departments of family medicine in Israel. The programs did not involve pharmaceutical companies.

For analysis, the family physicians were categorized into three groups: residents in family medicine; board certified family physicians (BCFP) who had completed 4 years of residency in family medicine and passed the final examinations; and non-board certified family physicians (non BCFP) who mostly did not have training in family medicine. All participating physicians were actively providing direct patient care during the survey period, and were affiliated with one of the 4 HMOs that provide primary health care to all Israeli persons. According to the websites of the HMOs, 6761 family physicians provide primary health care in Israel (Table 1).

**Table 1:** The number of surveyed participating family physicians (FPs) compared to the number of FPs in HMOs

	HMO 1	HMO 2	HMO 3	HMO 4
Israeli FPs (n = 6761)	3521 (52.1 %)	1368 (20.2 %)	985 (14.6 %)	887 (13.1 %)
Surveyed FPs (n = 362)	208 (57.4 %)	55 (15.2 %)	51 (14.1 %)	48 (13.3 %)

The survey was distributed, with no incentive offered to participants, and collected during the same session, so as to maximize respondents’ compliance. The survey focused on physician attitudes, sources of knowledge and recommendations regarding diabetes management, as well as attitudes toward people with type 2 diabetes. For example, the physicians were asked to select from a list, the recommendation they give their patients with diabetes; such as increasing physical activity and meeting with a dietitian. Physicians were also asked to respond “agree” or “disagree” to statements about diabetes medications, and their attitudes to their patients with diabetes. For example: “My patients with diabetes lack motivation and the willpower to change their lifestyle compared to my patients without diabetes.”

Respondents’ demographic and professional information, including age, gender, years in practice and professional status, was accessed. Physician’s responses to the survey were entered into the Windows Excel database and analyzed in SPSS for Windows. We used descriptive statistics to analyze the responses to each item. Chi-square testing was used for categorical variables, and analysis of variance for continuous variables.

This study was exempted from applying for approval from the institutional ethics committee, since no patients were involved.

## Results

A total of 458 family physicians were invited to participate in the survey at 18 CME programs. Of them, 362 (79 %) filled and returned the questionnaires. Age and seniority in family medicine differed significantly among the three groups of physicians; the residents were the youngest and the non BCFPs the oldest (Table 2). An almost equal number of male and female physicians participated in the study (51 % females). Seventy-five percent of family physicians work in urban clinics and only a minority (9 %) work in independent practices; 46 % reported the proportion of diabetes patients to be 5–9 % of all their patients above age 20 years and 47 % reported this proportion to be more than 9 %.

**Table 2:** Professional status, age and seniority (number of years in practice) of participating physicians

Professional status	All	Residents	Board certified family physicians	Non-board certified family physicians
No. of physicians	362 (100 %)	108 (31 %)	137 (34 %)	117 (35 %)
Age (mean ± SD)	43.8 ± 8.4	33.7 ± 4.8	43.4 ± 7.1	49.5 ± 7.4
Seniority (in years) (mean ± SD)	14.8 ± 9.2	2.6 ± 3.4	12.1 ± 6.5	21.2 ± 8.8

### Diabetes Management

Seventy-two percent of the respondents considered their role in influencing diabetes management as ‘very important’; 66, 15, and 30 % attributed much influence to family members, friends and the media, respectively. Fifty percent reported referring some of their patients, according to their disease severity and complications, as well as poorly controlled, insulin-treated patients for secondary care. About one-third (36 %) of the respondents manage the diabetes also of their patients who are treated with insulin; yet, 60 % reported lacking knowledge about nutritional issues. Relatively few (25 %) advocated group support meetings. A lower proportion of non-BCFPs than residents and than BCFPs recommend to their patients with diabetes, family counseling and group support meetings; and fewer provide nutritional counseling themselves (Table 3).

**Table 3:** Recommendation of diabetes management advised by family physicians (in percentage)

Recommendations	All N = 362 (%)	Residents N = 108 (%)	Board certified family physicians N = 137 (%)	Non-board certified family physicians N = 117 (%)	*P* value**
Increase physical activity	95	95	96	94	NS
Counseling on disease complications	81	87	84	76	NS
Family member support counseling	70	81	79	73	0.002
Referral to a dietitian	97	94	97	98	NS
Diet counseling by the family physician	71	82	79	60	0.001
Lifestyle change counseling by family physician	97	98	96	91	NS
Group support meetings	43	61	58	29	0.003
Weight loss counseling	96	93	97	91	NS
To generally eat less	91	92	94	86	0.001

Percentage of physicians who report giving the advice always/often

^**^ *P* value difference among sub-groups

### Acquisition of Knowledge of Diabetes Management

Eighty-six percent of the respondents considered their professional experience to contribute greatly to their knowledge and attitudes toward diabetes counseling, 68 % stated learning from medical journals and 62 % from professional colleagues. Fifty-six percent of the respondents regarded CME activities as an important contributor to their knowledge, while only 24 % stated that medical school contributed. Fifty percent of BCFPs and residents considered medical journals to contribute greatly to their professional knowledge.

### Attitudes Toward Patients with Diabetes

Physicians’ reactions to statements about patients with diabetes and the management of diabetes are presented in Table 4. Compared to residents and BCFPs, a larger proportion of non-BCFPs responded that their patients with diabetes have more difficulty in changing their lifestyle habits than do other people. A smaller proportion of non-BCFPs knew that GLP1 analog medications (i.e. liraglutide, exenetide) do not usually cause weight gain.

**Table 4:** Physicians’ attitudes to statements on diabetes patients—the percentage of family physicians who responded ‘agree’ (in percentage)

Statement	All N = 362 (%)	Residents N = 108 (%)	Board certified family physicians N = 137 (%)	Non-board certified family physicians N = 117 (%)	*P* value**
Patients with diabetes have more difficulties in changing their lifestyle than do other people	48	41	43	57	<0.001
Most patients with diabetes have low compliance in medication taking behavior compared to others	29	22	16	36	<0.001
Reducing the HBA1C value to under 6.5 % is not recommended for all age groups	79	85	89	56	<0.001
Accurate nutritional and caloric labeling of food would contribute to glucose control	71	70	69	67	NS
Insulin treatment is the most effective medication for reducing HbA1c level	59	62	65	54	NS
A problem with GLP1 analog treatments is that they cause weight gain	58	67	69	42	<0.004

^**^ *P* value difference between sub-groups

## Discussion

Primary care clinicians provide the foundation for health care systems. They serve as the central cohesive element that improves access, connects and bridges to secondary and tertiary care, and provides continuity for patients and families [10]. Diabetes management is especially challenging in the primary care setting due to the substantial resources and knowledge required. Nevertheless, only 9 % of family physicians who responded to the survey reported that they usually refer their patients with diabetes to secondary care.

BCFPs and their residents tended to be younger, and to exhibit greater knowledge of diabetes than did non-BCFPs. Another Israeli study reported a correlation between the evidence-based knowledge of primary care physicians and the quality of care they provide [14]. Further, a study of family physicians who treat patients with diabetes in Saudi Arabia found that those with fewer years of experience scored higher on measurements of overall knowledge, attitudes and practice than did those with a longer duration of practice [15].

The majority of respondents to the current survey answered that family physicians contribute greatly to the management of their patients’ diabetes management. Patients’ family members were also considered to have high influence. A study recently conducted by Kaiser Permanente found that adults with diabetes who perceive family members’ behavior as unsupportive are less adherent to their medication regimen [16].

The recommendation by most primary care providers to engage in physical activity concurs with the increasing evidence of the importance of aerobic exercise in diabetes management [17–19]. Also, 97 % of the respondents to the survey reported advising weight loss for obese patients and referring to a dietitian. The Israeli healthcare system provides free access for individuals with diabetes to a dietitian. In contrast, heavy workload make in-depth and successive dietary counseling by the family physician infeasible. Only one quarter of the respondents to the current survey reported advising their patients about group counseling sessions. More BCFPs reported advising patients to participate in group support meetings than did non-BCFPs and residents. Group visits have been shown to improve self-efficacy and glycemic control [20]. However, a study that compared the effects of group-based versus individual-based diabetes self-management programs on health-related quality of life reported no statistically significant differences between the approaches [21].

Almost half of the respondents, including a significantly higher proportion of non-BCFPs than BCFPs and residents, stated that their patients with diabetes have more difficulty making lifestyle changes than do their patients without diabetes. A number of studies have documented stereotypical attitudes toward patients with diabetes held by health professionals, including family physicians [22, 23].

Altogether, 42 % of the respondents, more non-BCPs than BCFPs and residents, answered incorrectly regarding adverse effects of the GLP-1 receptor analogue. A secondary analysis of studies conducted among various populations concluded that family physicians lacked knowledge of the exact mode or mechanism of action of medications they prescribe; notably, regarding new treatment strategies recently added to existing guidelines [24]. An area for future research is exploration of the effects of gaps in knowledge on diabetes management, including prescriptions of new treatments.

Insulin is considered one of the most effective therapies for achieving glycemic goals [25]. In the current study, 59 % of all family physicians responded that insulin is the most effective treatment for reducing HbA1c levels. In Hayes et al.’s study [26] the majority of family physicians agreed that the benefits of using insulin to prevent or delay complications outweighed the risks of hypoglycemia and weight gain for most patients; though consensus was lower for severely obese and elderly patients.

One-third of the respondents to our survey refer patients who need insulin to diabetes specialists. In their secondary analysis of studies conducted around the world, Murray et al. [24] concluded that family physicians do not feel competent in administering insulin therapy, lack knowledge of when to initiate it, and have difficulties in maintaining and adjusting it. Hayes et al. [26] reported that two-thirds of family physicians consider the initiation of insulin as one of the most difficult aspects of managing their patients with type 2 diabetes, and confirmed that family physicians in the US do not agree about aspects of insulin initiation. Consensus was reached, however, regarding the risks and benefits of insulin, and patient fears about initiating insulin [26].

Distribution of the questionnaire before a CME session helped to achieve a high response rate. However, it may have resulted in a selection bias, since all respondents were physicians who were interested in enriching and updating their knowledge though formal CME sessions. Assessing the knowledge and attitudes of family physicians who do not regularly attend CME conferences is also important. The use of an open-ended questionnaire and interview are additional means that should be used to access more in-depth information regarding the knowledge, attitudes and needs of family physicians in the care of individuals with diabetes. In conducting the survey, we encountered difficulties in persuading the family physicians to fill the questionnaires. CME program local leaders assisted, and we ultimately achieved a relatively high response rate. The differences observed in responses according to professional background may be unique to Israel, where most non-BCFPs are physicians who emigrated from the former USSR in the early 1990s. We were unable to find, for comparison, research papers that dealt with attitudes toward and knowledge of diabetes issues among family physicians according to their professional background. An important subject for investigation is the degree that physician responses correlate with their behaviors.

## Conclusions

The findings of this survey highlight the weight and challenge of diabetes management in the primary care setting. While most family physicians in Israel manage diabetes for the majority of their patients with this disease, gaps in resources and knowledge exist. The implementation of strategies that will enhance the competencies and confidence of family physicians in diabetes management are important for achieving successful treatment.
